# Introducing pathways to resilience in the Karamoja Cluster

**DOI:** 10.1186/s13570-021-00214-4

**Published:** 2021-11-24

**Authors:** Andy Catley, Elizabeth Stites, Mesfin Ayele, Raphael Lotira Arasio

**Affiliations:** Feinstein International Center, Friedman School of Nutrition Science and Policy, Tufts University, P.O. Box 6934, Kampala, Uganda

## Introduction

**Fig. 1:**
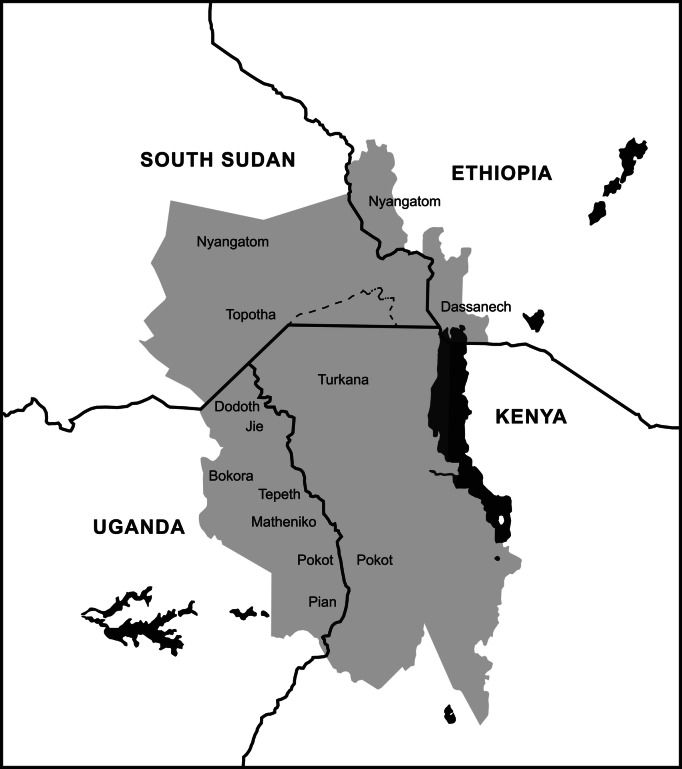
Map of the Karamoja Cluster

The Intergovernmental Agency on Development defines the Karamoja Cluster (Fig. 1) as a cross-border region comprising southwest Ethiopia, northwest Kenya, southeast South Sudan and northeast Uganda, occupied by at least 13 pastoralist and agro-pastoralist communities (Intergovernmental Agency on Development 2021) with ethnic, linguistic and cultural similarities. These ethnic groups include the Bokora, Dessenech, Didinga, Dodoth, Jie, Matheniko, Nyangatom, Thur, Pian, Pokot, Tepeth, Topotha and Turkana (Gray et al. 2003), with common borders of about 8,400 km. Although the Karamoja Cluster can also be characterized by its physical isolation, under-development and conflict, different areas of the Cluster have very different political and conflict contexts. These vary from the relative stable and emerging local governance and policy environment in Turkana and Pokot in Kenya, albeit with persistent livestock and localized livestock raiding, to the severe political instability and widespread armed conflict in South Sudan.

The Karamoja region in northeast Uganda has long been characterized as under-developed and insecure, with repeated episodes of violent livestock raiding within Karamoja itself, and affecting neighbouring areas in Kenya, South Sudan and Uganda. Since the late 1970s, ‘modern’ forms of raiding evolved using automatic weapons, causing considerable loss of human life (Gray et al. 2003). The Uganda government’s response to this violence included a series of disarmament campaigns, and between 2006 and 2010, disarmament was led and enforced by the Ugandan army. This was a particularly dark period in Karamoja’s recent history, with armed air and ground attacks on civilians, frequent reports of human rights abuses and the introduction of ‘protected kraals’, controlled by the army. The containment of livestock in these kraals disrupted access to pasture and water and led to atypical outbreaks of diseases; the net outcome was a marked decline in livestock productivity and survival, and corresponding negative impacts on livelihoods and human nutrition. Before this disarmament campaign, Karamoja already had the worse human development indicators in Uganda.

From 2011, it became evident that the disarmament campaign had created a secure environment in Karamoja while also severely undermining livelihoods. The post-disarmament period saw a new influx of international aid organizations and programmes, as well as some improvements to infrastructure and services, and market and business activity. By 2016, the ten main aid donors in Karamoja had committed funding to development programmes valued at US$95.2 million for 2017 (Karamoja Resilience Support Unit 2016a), and 59 non-governmental organizations (NGOs) were operational (Karamoja Resilience Support Unit 2016b). Starting in early 2016, the Karamoja Resilience Support Unit (KRSU) aimed to support evidence-based programming and policies in Karamoja across all sectors and provide coordination support to a multi-donor platform called the Karamoja Development Partners Group. The KRSU conducted technical reviews, studies and analyses, and provided direct technical support to central and local government, aid donors and NGOs, while also tracking research, programmes and policies in neighbouring areas of Kenya and South Sudan.

By mid-2018, the KRSU recognized a familiar set of livelihoods’ challenges and opportunities in Karamoja. For example, in 2017, it was evident that livestock markets were dynamic and performing well, with the region supplying various internal markets in Uganda, as well as cross-border trade into Kenya and South Sudan (Aklilu 2017). Yet despite this buoyant economic activity, bi-annual surveys by the Government of Uganda and UN agencies showed no clear improvement in human food security or nutrition indicators. Other research suggested that an important effect of the disarmament programme was to encourage a concentration of livestock ownership among wealthier owners (e.g., Stites et al. 2016), while more poorer herders were pushed into non-livestock livelihood activities that generated limited income despite considerable effort (Bushby and Stites 2016; Iyer and Mosebo 2017). These trends indicated that a ‘Moving Up-Moving Out’ pattern of livelihoods’ change was occurring in Karamoja and in a comparable way to pastoralist areas in other countries (e.g., Catley and Aklilu 2013). In summary, households with enough animals engage pro-actively in livestock markets while retaining a sufficient herd for milk production, financial capital and growth; households with few animals struggle to acquire the minimum number needed for basic household food security and become caught in a cash and livestock poverty trap.

## The Pathways to Resilience in the Karamoja Cluster conference

To better understand these trends and other issues affecting livelihood options in Karamoja (Uganda) and the wider Cluster, the KRSU organized Karamoja’s first international research conference Pathways to Resilience in the Karamoja Cluster, in Moroto in May 2019. The call for conference papers was broad in scope and allowed for submissions related to any sector but with a clear and direct link to livelihoods and resilience. Papers could be original research, reviews or project evaluations, especially evaluations showing livelihoods’ impacts. The conference called for papers from the ‘Karamoja Cluster’ and within this regional framing was the notion of towns such as Moroto in Karamoja (Uganda) and Lodwar in Turkana (Kenya) being regional hubs and connected, cross-border centres of economic activity and trade. This framing drew heavily on the international conference on “The Future of Pastoralism in Africa” in Addis Ababa in 2011 (Catley et al. 2013) and recognized the stability and optimism in Karamoja in the post-disarmament period and the growing social and economic ties between Karamoja and Turkana. Within the Karamoja Cluster, the conference aimed to highlight two inter-linked dimensions of livelihoods and development viz. gender and diversification. The gender dimension took account of research on human nutrition and ecology in Turkana from the early 1980s (e.g., Galvin 1985) and Karamoja from around 2008 (Gray et al. 2008), as well as more recent research in Karamoja on the severe nutritional and livelihoods’ stresses on women related to loss of livestock, violent livestock raiding and the persistence of domestic gender-based violence (Stites and Mitchard 2011; Stites and Howe 2019). On diversification, we recognized the considerable body of existing research on this topic and the notion of diversification in pastoralist contexts often being either positive or negative. After Little (2016), positive (or adaptive) types of diversification ‘include activities that improve incomes, welfare, and resilience to shocks without damaging the environment and/or conflicting with the predominant livelihood (pastoralism)’. In addition, positive diversification avoids social disruption, is relatively safe and does not expose individuals to physical or sexual abuse; it also produces income that is meaningful relative to household needs. In contrast, negative (maladaptive) diversification can result in environmental damage, societal change or activities that undermine pastoralism. It often leads to very limited income, e.g. from causal labour, or exposes people to unsafe or abusive working conditions.

After review of paper submissions, 32 papers were selected for presentation and these were grouped into nine thematic areas: livestock and livelihoods (five papers); peace and conflict (four papers); mobility, land and water (four papers); resilience, risk and change (three papers); diversified livelihoods (three papers); human nutrition (five papers); education and health (three papers); emergency and extractives (two papers); and programming experiences (three papers). The papers were presented over 3 days and were preceded by three keynote presentations.

## Papers in the Special Issue

This Special Issue of Pastoralism presents selected papers from the conference, preceded by a commentary that was drawn from the opening keynote presentation (Darlington Akabwai, this issue). As expected, livestock and issues related to the role of livestock in livelihoods and livestock management were featured heavily in the conference. In a second keynote presentation, Frank Muhereza provided an overview of recent livestock and pastoralism policies in Ethiopia, Kenya, South Sudan and Uganda and noted the mixed policy narratives around pastoralism but a constant thread of commercialization ambitions by national governments in contexts of continuing conflict and contested access to land (Muhereza 2019). Where livelihood diversification is positive, leading to meaningful and sustained income, it is most often associated with livestock in some form. The issue of declining per capita ownership of livestock in Karamoja and skewed ownership towards wealthier households was covered by Andy Catley and Mesfin Ayele (this issue). They reported that 47% of households in Karamoja owned only 1.2 Tropical Livestock Unit (TLU)/capita or less, against an estimated threshold of 3.3 TLU/capita for viable agro-pastoralism. Against the conference theme of pathways to resilience, this paper proposed that these pathways for poorer households remain highly uncertain. Despite declining livestock ownership for many, Padmini Iyer (this issue) describes how social networks and obligations in Karamoja centred on livestock friendships and remained active and important during normal times and times of crisis. In a broader sense, this paper indicates how many people were able to survive with so few livestock and with limited positive diversification opportunities.

The third keynote presentation focused on gender and livelihoods in Karamoja and how changes in livestock ownership led to corresponding changes in the roles of women and in particular, an increasing domestic burden in terms of household incomes and diets (Stites 2019). The gendered effects of sedenterization, livestock commercialization and migration were also discussed. Related to both gender and diversification, Padmini Iyer and Elizabeth Stites (this issue) examined the controversial practice of alcohol production in Karamoja and unpacked and compared the well-established practice of brewing beers from local cereals to a recent surge in the consumption of cheap ‘hard liquor’ with very high alcohol content. They describe the societal impacts of this consumption and the reasons behind it, including severe livelihoods’ pressures, loss of livestock and, especially for men and male youths, loss of identity. In their paper on sexual and reproductive health of adolescent girls in Karamoja, Stella Achen and colleagues (this issue) position reproductive health risks within the wider context of culture and behaviour, and notably, the practices of early marriage and bridewealth payments. As per capita livestock ownership declines, poorer families regard the marriage of young daughters as a convenient way to acquire livestock, and so health risks for girls are closely associated with livelihood trends.

Two papers in the Special Issue describe the changing livelihoods of pastoralists and their engagement with the state and development actors. In Ethiopia’s lower Omo valley, Fana Gebresenbet (this issue) associates a decline in the food security and livelihoods of Bodi agro-pastoralists to a large-scale government irrigated sugar plantation, developed near the Gibe III dam from the early 2010s. In this case, increasing market engagement by the Bodi was mainly in the form of distress sales, which contrasted with the local government views of this engagement as a positive development. In Turkana County in northwest Kenya, Gregory Akall (this issue) focuses on the development impacts and issues around small-scale irrigation from the mid-1960s. He describes an increasing array of pressures on secure access to land by pastoralists and, as livelihoods declined, shifts towards negative forms of diversification, especially for women and girls.

Against the myriad of aid organizations, and development and humanitarian programmes in the region—especially in Karamoja and Turkana—Cory Rodgers (this issue) examines the quality of the interaction between outsiders and pastoralists, and the specific issue of customary land tenure in a refugee resettlement scheme in Turkana. He argues that despite community participation being widely accepted in a rights-based approach to development, meaningful participation was limited and the aid organization in question relied on dialogue with urban professionals and politicians. The key finding that this organization had limited understanding of local context or limited capacity to engage directly with pastoralists resonates loudly across other areas of the Karamoja Cluster.

## Research gaps and emerging issues

The Pathways to Resilience conference in Karamoja took place in a period of relative peace and stability, and a general sense of optimism that the region could emerge finally from decades of armed violence, and political and economic isolation. At the same time, the conference papers clearly described the challenges ahead for resilient livelihoods in the Karamoja Cluster, especially for households living on the edges of pastoralism or no longer owning livestock. In common with other pastoralist areas of East Africa, the Cluster is characterized by substantial numbers of households who are livestock-poor and cash-poor and who make ends meet through mixes of local social support, negative diversification activities and, in some areas, social protection programmes. Where innovation occurs, it is often among those with sufficient wealth, e.g. the division of herds into a ‘herd for the market’ and a ‘herd for milk and savings’. The pathways to resilience for women and girls are especially uncertain, and education might be seen as a critical element for providing access to better paid and safer employment, beyond activities such as charcoal production, brewing or causal labour. Yet notably, we received no papers that described educational approaches or models in the Cluster that demonstrated improved education for girls or boys, that considered the need to adapt conventional schooling to the pastoralist context, or which assessed the affordability of education for poorer households. In 2016, in Karamoja (Uganda) alone, there were 20 aid organizations implementing or supporting 29 education projects (Karamoja Resilience Support Unit 2016b).

In addition to more research on delivering effective and affordable education, other research opportunities included growing trade and social ties across borders, such as between Karamoja and Turkana, and the flows of goods, services and people between towns such as Moroto and Lodwar. Whereas issues of land governance and tenure have been well-documented and openly discussed for Turkana, the issue of land access in Karamoja remains under-researched and few, if any, aid programmes work directly on the policy and institutional arrangements that are needed to secure access to productive rangeland. There are many concerns in the Karamoja case, with for example, nearly 25% of land in Karamoja being subject to government-issued exclusive mineral exploration and location licences, that were reportedly shrouded in anonymity (Rugadya et al. 2010). In Ethiopia, one conference presentation showed that even the most remote areas are not immune to massive land acquisition by the state, with the declining livelihoods among Bodi agro-pastoralists in South Omo in the late 2000s (Gebresenbet, this issue) being comparable to displacement and destitution of Ittu and Kerrayu Oromo pastoralists in the middle Awash Valley of Ethiopia from the early 1960s (Carr 2017).

Since the 2019 conference and during the preparation of this Special Issue, the pastoralist areas covered by the conference were affected by COVID-19 restrictions, and in the Karamoja case, people faced an unprecedented mix of market closures, movement restrictions, price rises and hindrances to livestock and crop production, with serious impacts on food security and livelihoods (Arasio et al. 2020). But perhaps more alarming has been the re-emergence of modern weapons and livestock raiding in Karamoja and the concern that the economic and security gains seen during the immediate post-disarmament period will disappear.

## Data Availability

The paper is a commentary and uses no data.
